# Telling partners about chlamydia: how acceptable are the new technologies?

**DOI:** 10.1186/1471-2334-10-58

**Published:** 2010-03-09

**Authors:** Carol A Hopkins, Meredith J Temple-Smith, Christopher K Fairley, Natasha L Pavlin, Jane E Tomnay, Rhian M Parker, Frank J Bowden, Darren B Russell, Jane S Hocking, Marcus Y Chen

**Affiliations:** 1 Melbourne School of Population Health, The University of Melbourne, Carlton, Victoria, Australia; 2 Melbourne Sexual Health Centre, 580 Swanston Street, Carlton, Victoria, Australia; 3 Melbourne Graduate School of Education, Old Geology Building, The University of Melbourne, Carlton, Victoria, Australia; 4 Centre of Excellence in Rural Sexual Health, School of Rural Health, The University of Melbourne, 49 Graham Street, Shepparton, Victoria, Australia; 5 Australian Primary Health Care Research Institute, Australian National University, Canberra, ACT, Australia; 6 Australian National University and Canberra Sexual Health Centre, Garran, ACT, Australia; 7 Cairns Sexual Health Service, Cairns Base Hospital, Cairns, Queensland, Australia; 8 Key Centre for Women's Health in Society, The University of Melbourne, Carlton, Victoria, Australia

## Abstract

**Background:**

Partner notification is accepted as a vital component in the control of chlamydia. However, in reality, many sexual partners of individuals diagnosed with chlamydia are never informed of their risk. The newer technologies of email and SMS have been used as a means of improving partner notification rates. This study explored the use and acceptability of different partner notification methods to help inform the development of strategies and resources to increase the number of partners notified.

**Methods:**

Semi-structured telephone interviews were conducted with 40 people who were recently diagnosed with chlamydia from three sexual health centres and two general practices across three Australian jurisdictions.

**Results:**

Most participants chose to contact their partners either in person (56%) or by phone (44%). Only 17% chose email or SMS. Participants viewed face-to-face as the "gold standard" in partner notification because it demonstrated caring, respect and courage. Telephone contact, while considered insensitive by some, was often valued because it was quick, convenient and less confronting. Email was often seen as less personal while SMS was generally considered the least acceptable method for telling partners. There was also concern that emails and SMS could be misunderstood, not taken seriously or shown to others. Despite these, email and SMS were seen to be appropriate and useful in some circumstances. Letters, both from the patients or from their doctor, were viewed more favourably but were seldom used.

**Conclusion:**

These findings suggest that many people diagnosed with chlamydia are reluctant to use the new technologies for partner notification, except in specific circumstances, and our efforts in developing partner notification resources may best be focused on giving patients the skills and confidence for personal interaction.

## Background

Ensuring the partners of those diagnosed with bacterial sexually transmitted infections are notified, tested and treated is a well established strategy in limiting the transmission of these infections [1]. Chlamydia-infected patients are commonly encouraged by health care providers to contact their partners themselves. While this is a relatively simple and inexpensive strategy, it has been estimated that only 40-60% of partners are actually contacted this way [2-4], suggesting the need for innovative strategies to increase the number of partners being notified.

In particular, there has been growing interest in the role of the new technologies such as SMS, email and the internet in enhancing partner notification [5-9]. In recent years, innovative websites have offered facilities for sending personal or anonymous emails and text messages to partners [10-13]. To date, however, there has been limited research into the acceptability of these methods for partner notification [14,15].

This study aimed to determine the methods used by participants to contact their partners, the reasons for choosing those methods, and their opinions of various partner notification methods including those utilising these new technologies.

## Methods

Semi-structured, telephone interviews were conducted with people who were recently diagnosed with chlamydia at three sexual health centers and two general practices in Australia between November 2006 and May 2007. The sexual health centers were located in Melbourne (Victoria), Canberra (Australian Capital Territory) and Cairns (Far North Queensland) while the two general practices were from rural Victoria. Ethics committee approval was obtained from the University of Melbourne and the Alfred Hospital research ethics committees.

Doctors and nurses at participating centres asked people who tested positive for chlamydia if they would agree to being contacted by a research nurse to discuss the study. The research nurse then phoned the person, explained the study, obtained verbal consent and arranged a time to conduct a 30-40 minute telephone interview with them. While the interview explored a range of issues relating to partner notification, this paper reports only on the findings regarding partner notification methods. People were excluded from the study if they did not speak English, were under 18 years of age, or if they had already been told by a partner that they were at risk of chlamydia. We did not ascertain whether participants believed they had been infected by partners or whether they had exposed partners to chlamydia.

The interviews were audio-taped, transcribed and coded for emerging themes using NVIVO (V7.0), a qualitative research software program. The coding was conducted independently by two researchers (CH and MT-S) and then discussed to achieve consensus on common themes.

## Results

### Characteristics of the sample

A total of 66 people were invited to take part in the study and 55 agreed. Of these, 15 were not able to be contacted for interview, despite at least three attempts. In all, 40 people were interviewed - 15 from Victoria, 15 from Queensland and 10 from the Australian Capital Territory. Thirty-eight were from sexual health centres and 2 from general practices.

Of the 40 people interviewed, 62% (25) were female and 38% (15) were male. Sixty-five percent were in the 18-25 year old age group (range 18-55 years). In terms of education, 58% (25) had secondary education only, 7% (3) had a trade qualification and 35% (14) had a tertiary degree or diploma. Eighty-eight percent (35) of the participants were heterosexual. Four interviewees were men who have sex with men (MSM) and 1 man was bi-sexual. While numbers of sexual partners in the preceding 6 months varied from 1 to 40, almost three-quarters of the sample (29) had less than 5 partners over that time.

### Methods used to tell partners about chlamydia

Overwhelmingly, participants contacted their partners either in person or by phone. Of the 36 participants who contacted at least one partner, 56% (19) did so face-to-face and a further 44% (15) used the phone. Some of those who used the phone wanted to tell their partners in person but were unable to because of circumstances. Only 17% (6) chose to use the newer technologies of SMS and email and 11% used the services of health department contact tracers. A number of participants used multiple methods to contact their partners.

### Reasons for choosing methods

Those who chose to tell their partners face-to-face frequently stated that it was the only appropriate way to talk with someone about a personal, sensitive issue such as having chlamydia.

It seemed like the right thing to do. I think he deserved for me to tell him with him there and not just call him up. (female, 20, ACT)

Being able to see their partner's reaction, as well as demonstrating respect and consideration, was also important to many interviewees.

...I felt more comfortable that I could see their reaction and it was just more courteous to tell them to their face. (female, 21, Vic)

Those who told their partners by phone commonly mentioned speed and convenience as the main reasons for choosing this method.

*It was the quickest and most convenient way at the time. As soon as I found out I wanted to let people know straightaway. (male, 24, Vic*)

Where infidelity was suspected, the phone provided a means to

"get it out of the way and find out what was going on instead of having to wait." (female, 23, Vic)

For some, avoiding direct contact with the person was a consideration in choosing phone, email or SMS.

I hadn't seen him in a long time and hadn't spoken to him and wasn't really confident seeing him and had to have a little bit of distance in the conversation. (Phone) (female, 25, Vic)

I didn't really know the person all that well and I didn't particularly like him and so it was easier to get it out of the way (SMS). (male, 24, Vic)

Interviewees who used email often did so because their partners were overseas or elsewhere on business. SMS was sometimes chosen when partners were not answering their mobile phones. Several participants used SMS to ask their partner to call them and only delivered the news about chlamydia when speaking with them. Another used SMS to send the details of an STI clinic.

I sent her a text message saying, "I need to catch up. I have something to say." And then she called back demanding what was wrong and I just sort of told her over the phone. (male, 20, Vic)

Some participants chose to use health department contact tracers for partner notification because they wanted to avoid contact with their partner, often because they feared their reaction. For one, avoiding public embarrassment and shame was also an important factor.

And like I said before, it (having chlamydia) would put my character down and people would be calling out, 'You've got some sore blah, blah, blah.' They would call you everything under the sun. They would call you every name. It's a small town, people know. (male, 27, QLD)

### Opinions of contact methods

In discussing their views of different methods of partner notification for chlamydia, interviewees made a clear distinction between traditional, personal methods of contact such as face-to-face and phone and the newer, less personal methods of email and SMS. Generally, people believed that a sensitive, personal issue such as having a sexually transmissible infection needed to be discussed personally with the partner. However, SMS and email were seen to be appropriate, useful or even advantageous depending on the circumstances.

### Face-to-face

Almost all interviewees believed face-to-face was the "gold standard" in partner notification because it demonstrated respect, consideration and caring for the partner. Interviewees particularly believed partners would think better of them for telling them this way.

I think it's the only way to go. And they think more of you and they commend you, really, even though you've given them an STD. (male, 21, ACT)

Being able to see their partner's reaction and offer appropriate support was another advantage frequently mentioned by interviewees.

By doing it face-to-face, you can see their reactions more and judge how they are feeling about it. And if you can judge their reactions or their body language you can sort of say the things you need to make them feel better about the situation as well. (female, 18, Vic)

When pressed for the negatives of face-to-face communication, most interviewees mentioned feeling nervous, awkward and embarrassed.

You've just got to be very brave to do it. Having to talk to someone about such a big issue is very hard. (female, 23, Vic)

Fear of the partner's angry or derogatory reaction was an issue for some.

I wouldn't want to do that. ...I'd feel dirty standing there telling them, "Oh, by the way, I have Chlamydia and you have to get yourself checked out". They'd stare me up and down and say, "That's gross". (female, 19, ACT)

### Phone

In situations where face-to-face communication was not possible or desirable, phone was seen by most interviewees as a reasonable alternative for telling partners about chlamydia. Speed and convenience were viewed as particular advantages of this method.

I can do it straight away. As soon as I find out I can give them a call. I don't have to make arrangements to meet them somewhere and take time out of their day just so I can tell them something. (male, 24, Vic)

I wasn't looking forward to it and so I just got it out of the way, straight away. (male, 27, Vic)

Others acknowledged that making a phone call is less risky, confronting and embarrassing, particularly because it offers more distance and control.

There's a sense of anonymity where you don't have to worry so much about the person's reaction. You can hang up the phone and it's over and you don't have to deal with it any further than that. (female, 25, Vic)

Some felt that using the phone was cowardly.

It's really the 'chicken' way to tell someone. It's like telling him "It's over" (the relationship) over the phone. (male, 31, ACT)

Others regretted being unable to see their partner's reaction.

While you can hear what the person is saying, you can't really see their reaction as much as if you were face-to-face. You don't get a picture of how they feel. (female, 18, QLD)

One participant had a very practical objection to using his mobile phone to tell partners.

*Over the phone would be expensive 'cos it would take me a good half-hour, you know. That's like $30 or something. (male, 21, ACT*)

### Email

Generally, email was viewed as a poor option for telling partners about such a personal and sensitive matter as having chlamydia. Nevertheless, some acknowledged that email could be appropriate in certain circumstances.

Obviously there are better ways to tell someone, more honest ways. I guess it's kind of like breaking up with someone. But if that's the only option then I think it's a big step alone just to tell them. (female, 18, QLD)

Certainly email was seen as a better alternative to SMS because

*"you can at least be informative and provide links to show them where to go". (female, 19, ACT)*.

Another felt that she would be able to communicate more calmly and clearly by email.

You tend to babble if you are telling people bad news and you can be really systematic and outline what's happened and what needs to happen by email. (female, 24, QLD)

However, for most, the negative aspects of email outweighed the positives. As well as being seen as "impersonal", "rude", "gutless", "distant" and "uncaring", email communication was viewed as risky because it could be shown to other people.

I think a negative with both SMS and email is that anyone could see it. I don't think it's private. I wouldn't risk anybody else seeing it or showing his mates and saying, "Look at what this chick sent me." (female, 22, ACT)

Other negatives mentioned by interviewees included not being sure if or when the email reached the recipient, not having email addresses for partners and not having access to a computer and/or email facilities. One interviewee also felt that email communication would not be taken seriously.

It (email) is so informal. I think if you're going to tell somebody you have an STI you need to show a good enough level of respect to tell them in person, especially if they are going to take you seriously and go and get treated. (female, 18, Vic)

### SMS

Although acknowledged as being better than nothing, most interviewees believed that SMS was not a good way to tell partners about chlamydia. SMS was seen as being worse than email because of the brevity of the message.

I think it would be better to say it via the phone or face-to-face, but I think it's tough enough to tell someone as it is, and if your only way of telling someone is by SMS then at least you've told them. (female, 18, QLD)

Others felt SMS was acceptable if the relationship was brief and superficial.

I would SMS someone if it was a one-night stand and I didn't really care about them. I would be just letting them know. (female, 25, Vic)

SMS also had the advantage of avoiding personal contact and shame.

It would be a lot easier. You don't have to hear the disappointment or disgust in their voice. (female, 20, Vic)

One gay man was very enthusiastic about the anonymous SMS facility that is available from some websites.

I think the SMS thing is the best thing I've heard. The gay community in Melbourne is very, very small and so it would be good to be able to send out an anonymous SMS. (male, 24, Vic)

However, the majority of participants were strongly opposed to using SMS to tell partners about chlamydia stating that it was a "cold", "impersonal" "rude", and "lame" form of communication. Some spoke of their own reactions to receiving an SMS about this issue.

I think if someone sent me something like that by SMS I'd think, "You gutless wonder." ... "You didn't even care enough to tell me over the phone". (male, 31, Vic)

I got dumped by SMS once and there is no way that anyone should tell anybody anything like this by SMS. I think it's highly impersonal. I think it's worse than doing it by email. (male, 39, Vic)

As with email, participants were concerned that text messages could be read by others.

Anyone can go through someone's phone and read it (the SMS) and be like, "Oh my God, that person is bad and they got it from this person," and then they start spreading rumours. (female, 22, QLD)

Others wondered if the message would be taken seriously.

The only thing that concerns me is the validity when it comes through to their inbox. Whether they would realise that it's for real or whether they would think it was a joke. (male, 24, Vic)

### Letters

Participants were asked for their views about informing partners by letter, either one they wrote themselves or one that was given to them by their doctor. Letters that people wrote themselves were regarded as a better option for telling partners than email or SMS because they were seen as being more personal.

"It's much more personal to get something hand-written from someone and I think that if you are not able to say it out loud but you want to be more caring then it's a very good option." (female, 25, Vic)

Nonetheless, many were clear that communicating by letter was only acceptable if the relationship was not close, the person was too embarrassed to talk to their partner, or they had no other means of contact. In these cases, most agreed it was better than nothing.

On the downside, interviewees described letters as being old fashioned, time-consuming, a bit of a 'cop out' and able to be read by others.

Something written down like that could actually be read by someone else. No-one really writes letters now, except thank you letters. (female, 19, ACT)

Using a letter from the doctor to tell a partner about chlamydia was seen as having advantages, especially if the relationship was casual, distant, strained or potentially violent. The authority and anonymity of a doctor's letter were particularly appealing.

I think that would be fantastic. It takes away the strain of the situation. It absolutely has some 'street cred'. And the person is not friggin' going to come and knock down your door and say, "You gave me chlamydia, you bitch." And in that way, they do know to get tested and I suppose it's their responsibility then. (female, 21, Vic)

Interviewees expressed relatively few concerns about this method of partner notification, although some did not know their partner's address. Some suggested that it might be dismissed as a prank, the recipient might be frightened and worried by the news and that it would be inappropriate in close relationships. None of the participants in the study, however, used letters to notify their partners.

## Discussion

Overwhelmingly, respondents in this study believed that personal methods of communication, such as talking to someone face-to-face or over the phone, were the best ways for telling a partner they might be at risk of chlamydia. This was because they believed a personal and sensitive issue, involving an intimate relationship with another person, deserved to be discussed.

By comparison, the new technologies such as email and SMS were thought by most to be less personal. The lower acceptability of email and SMS for partner notification has been reported in other studies [15-17]. Despite these, email and SMS were seen to be appropriate and useful in some circumstances. In one study, almost a third of people who had not contacted all their partners agreed that having access to web-based tools such as anonymous email and SMS would have encouraged them to contact more partners [15] and evaluation of services that facilitates the sending of electronic cards, SMS and email to sexual partners have shown substantial uptake [10,13].

Interestingly, although letters were seen as outdated and irrelevant by most respondents, they were generally regarded as a better method than the new technologies for telling partners about chlamydia. A letter from the infected person, particularly if it was hand-written, was still seen as being personal, and a letter from a doctor had the advantage of authority and credibility. A preference for letters over emails and text messages has been reported in another study [16].

A frequently mentioned disadvantage of all written forms of communication - email, SMS and letters - was that they could become evidence of the person's infection with chlamydia. Respondents felt this could easily be shown to others, resulting in embarrassment and shame within their social group.

Most respondents expressed the view that the method of communication needed to match the relationship and the circumstances. Many were prepared to acknowledge that in the case of a brief, casual relationship, a relationship that had ended badly, or a relationship that was potentially violent, less personal methods of partner notification such as SMS, email or letter were acceptable. The same also applied to certain situations such as the partner being overseas or not answering their phone. In all these situations the general view was that it was better to tell the partner - by whatever means - than not to tell them at all.

A strength of this study was that the views on different methods of partner notification were obtained from individuals who had recently been diagnosed with chlamydia and who had confronted the issues around partner notification themselves. A limitation of the study was the limited number of participants in the study, in particular the low number of men who have sex with men (MSM) in the sample (5). It is possible that men in this group, who often have high numbers of casual or anonymous sexual partners, may be more receptive to the new technologies for partner notification. In a recent study, 81% of users of an MSM partner-seeking website reported they would be happy to be notified by email if they had been exposed to a sexually transmitted infection [14]. The findings of this study therefore may not be generalisable to MSM. Most participants were recruited from sexual health services, where partner notification is generally well recognized and supported. It is possible that the experiences and opinions of chlamydia-infected individuals may differ in situations where clinicians are less adept at discussing and managing for partner notification [18].

While most respondents used and favoured personal methods of communication for telling partners about chlamydia, it is important to remember that a proportion of people in the sample chose to use SMS or email to contact their partners. This suggests that the new technologies, although not generally accepted for partner notification to those in the study, still have an important role to play for some people. It should also be borne in mind that social desirability bias may have affected responses so that people were more likely to report favourably about personal methods over less personal ones.

## Conclusions

Most people prefer to tell their partners about their risk of chlamydia in person or by phone. The widespread availability of new technologies such as email and SMS does not necessarily mean that all people will use these methods for communicating about personal, sensitive issues such as having a sexually transmitted infection. Nonetheless, some people are choosing to use these new technologies for partner notification. To be effective in assisting patients with partner notification we need to take account of a range of personal preferences and circumstances. For most, this will involve giving people the information and skills to have difficult conversations with their partners, but for others it may mean providing easy access to template emails, text messages and letters that they can send either personally or anonymously.

## Competing interests

The authors declare that they have no competing interests.

## Authors' contributions

CAH recorded, transcribed and analysed all interviews and prepared the first draft of the manuscript. MJT-S contributed to the design of the study, assisted with analyzing interviews and developing themes and contributed to the drafting of the manuscript. CKF conceived the idea for the study, was the principle investigator on the funding grant and contributed to the design the study and drafting of the manuscript. NLP contributed to design of the study and interview schedule, assisted with recruitment of participants and reviewed the manuscript. JET contributed to the design of the study and themes explored. RMP contributed to the design of the study and the interview schedule. FJB managed the conduct of the study at the ACT site. DBR managed the conduct of the study at the Queensland site. JSH contributed to the design of the study and themes explored. MYC contributed to the design of the study, concepts and themes explored, reviewed the manuscript and oversaw the project. All authors have contributed to checking of the manuscript and approval of the final version.

## Pre-publication history

The pre-publication history for this paper can be accessed here:

http://www.biomedcentral.com/1471-2334/10/58/prepub
